# 3-Amino-1*H*-1,2,4-triazole-5(4*H*)-thione–4,4′-bipyridine (1/1)

**DOI:** 10.1107/S1600536812037671

**Published:** 2012-09-08

**Authors:** Qi-Ming Qiu, Yu-Han Jiang, Xu Huang, Qiong-Hua Jin, Cun-Lin Zhang

**Affiliations:** a Department of Chemistry, Capital Normal University, Beijing 100048, People’s Republic of China; b Key Laboratory of Terahertz Optoelectronics, Ministry of Education, Department of Physics, Capital Normal University, Beijing 100048, People’s Republic of China

## Abstract

The title two-component mol­ecular crystal, C_10_H_8_N_2_·C_2_H_4_N_4_S, was obtained unexpectedly by reaction of Zn(NO_3_)_2_·6H_2_O, NH_4_BF_4_ with 3-amino-1,2,4-triazole-5-thione (3-AMT) and 4,4′-bipyridine in water. The dihedral angle between the pyridine rings in the 4,4′-bipyridine molecule is 17.00 (13)°. In the crystal, N—H⋯N and N—H⋯S hydrogen bonds between the components lead to the formation of a three-dimensional network. Furthermore, the structure features face-to-face π–π stacking inter­actions between the 4,4′-bipyridine and triazole rings, with a centroid–centroid distance of 2.976 (2) Å.

## Related literature

For background to the 3-amino-1,2,4-triazole-5-thione ligand, see: Hao *et al.* (2010 ▶); Ma *et al.* (2008 ▶); Rakova *et al.* (2003 ▶). For related structures, see: Deng *et al.* (2005 ▶); Downie *et al.* (1972 ▶).
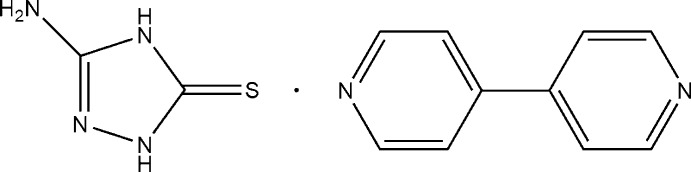


## Experimental

### 

#### Crystal data

C_10_H_8_N_2_·C_2_H_4_N_4_S
*M* *_r_* = 272.34
Monoclinic, 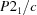

*a* = 13.5151 (12) Å
*b* = 6.9680 (5) Å
*c* = 16.5529 (14) Å
β = 122.385 (2)°
*V* = 1316.39 (19) Å^3^
*Z* = 4
Mo *K*α radiation
μ = 0.24 mm^−1^
*T* = 298 K
0.40 × 0.30 × 0.21 mm

#### Data collection

Bruker SMART CCD area-detector diffractometer
Absorption correction: multi-scan (*SADABS*; Bruker, 2007 ▶) *T*_min_ = 0.910, *T*_max_ = 0.951
6507 measured reflections
2315 independent reflections
1725 reflections with *I* > 2σ(*I*)
*R*_int_ = 0.034

#### Refinement

*R*[*F* ^2^ > 2σ(*F* ^2^)] = 0.040
*wR*(*F* ^2^) = 0.114
*S* = 1.09
2315 reflections
173 parameters
H-atom parameters constrained
Δρ_max_ = 0.40 e Å^−3^
Δρ_min_ = −0.29 e Å^−3^

### 

Data collection: *SMART* (Bruker, 2007 ▶); cell refinement: *SAINT-Plus* (Bruker, 2007 ▶); data reduction: *SAINT-Plus*; program(s) used to solve structure: *SHELXS97* (Sheldrick, 2008 ▶); program(s) used to refine structure: *SHELXL97* (Sheldrick, 2008 ▶); molecular graphics: *SHELXTL* (Sheldrick, 2008 ▶); software used to prepare material for publication: *SHELXTL*.

## Supplementary Material

Crystal structure: contains datablock(s) global, I. DOI: 10.1107/S1600536812037671/ff2081sup1.cif

Structure factors: contains datablock(s) I. DOI: 10.1107/S1600536812037671/ff2081Isup2.hkl

Supplementary material file. DOI: 10.1107/S1600536812037671/ff2081Isup3.cml

Additional supplementary materials:  crystallographic information; 3D view; checkCIF report

## Figures and Tables

**Table 1:** Hydrogen-bond geometry (Å, °)

*D*—H⋯*A*	*D*—H	H⋯*A*	*D*⋯*A*	*D*—H⋯*A*
N1—H1⋯N6^i^	0.86	1.98	2.828 (3)	171
N2—H2⋯N5^ii^	0.86	2.00	2.829 (3)	163
N4—H4*A*⋯N3^iii^	0.86	2.33	3.151 (3)	159
N4—H4*B*⋯S1^iv^	0.86	2.82	3.424 (2)	129

Symmetry codes: (i) 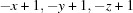; (ii) 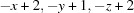; (iii) 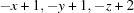; (iv) 
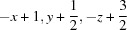
.

**Fig. 1.**
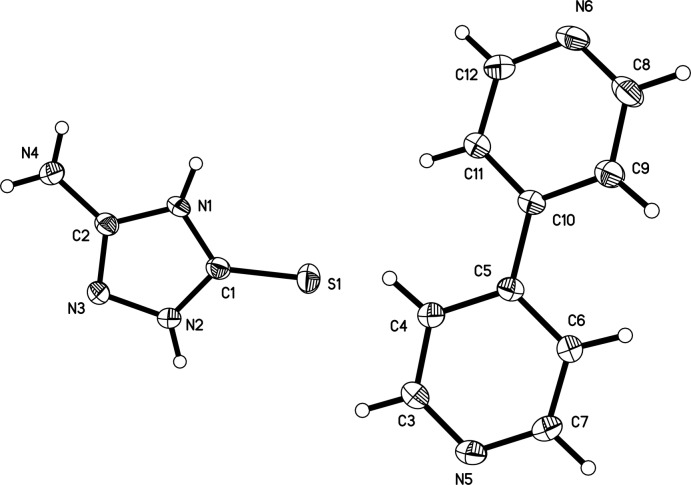
The molecular entities of the title compound, showing the atom-numbering scheme and with displacement ellipsoids drawn at the 50% probability level.

**Fig. 2.**
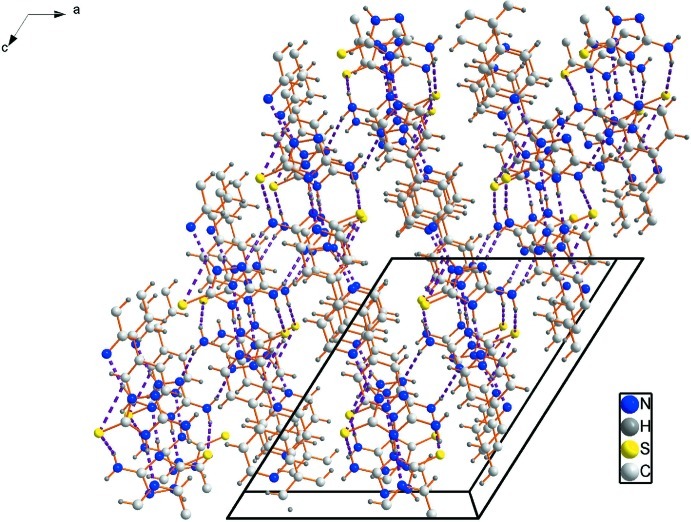
Three-dimensional structure formed by Intermolecular N—H···N and N—H···S hydrogen bonds.

## supplementary crystallographic information

### Comment

Supramolecular compounds have received much attention due to their structural diversities and potential applications as new materials. The ligand 3-amino-1,2,4-triazole-5-thione(3-AMT), with one –SH, one –NH_2_ group and three potential coordination nitrogen atoms of triazole, is excellent in building metal-organic supramolecular structures (Hao *et al.*, 2010; Ma *et al.*, 2008; Rakova *et al.*, 2003). Moreover, its nitrogen atom and sulfur atom may be involved in hydrogen bonding (Deng *et al.*, 2005; Downie *et al.*, 1972).

The title compound was unexpectedly obtained in the course of synthesizing 3-AMT-Zn(II) complexes. The molecular structure of the title compound is shown in Fig.1. The crystal structure of C_2_H_2_N_4_S.C_10_H_8_N_2_ has been determined at room temperature. The compound has the space group P2(1)/c, with a = 13.5151 (12), b = 6.9680 (5), c = 16.5529 (14) Å and beta = 122.385 (2)°. The hydrogen bonds are formed between the N—H donors of 3-AMT and the nitrogen atoms from 4,4'-bipyridine and 3-AMT, and the atoms N6, N5 and N3 act as acceptors with d(N1···O6) = 2.828 (3) Å, d(N2···N5) = 2.829 (3) Å, d(N4···N3) = 3.151 (3) Å, N1—H1···N6 =171°, N2—H2···N5 =163° and N4—H4A···N3 =159°, which are just similar to the corresponding distances and angles in the compound of C_10_H_8_N_2_.2C_2_H_3_N_3_S_2_ (Deng *et al.*, 2005). Intermolecular hydrogen bonds N—H···N and N—H···S between the components lead to the formation of a three-dimensional network (Fig.2). Furthermore, the structure is stabilized by the face to face π-π stacking interactions between 4,4'-bipyridine and triazole ring, with the centroid-centroid distance of 2.976 (2) Å.

### Experimental

A mixture of 3-AMT (0.6 mmol), NaOH (0.6 mmol), NH_4_BF_4_ (0.6 mmol), Zn(NO_3_)_2_.6H_2_O (0.3 mmol), 4,4'-bipyridine (0.3 mmol) and water (10 mL) was placed in a Teflon-lined stainless steel vessel (20 mL) and heated at 150°C for 72 h and then cooled to room temperature at a rate of 5°C/h.The filtrate was evaporated slowly at room temperature for 2 weeks to yield yellow crystalline products.

### Refinement

The final refinements were performed by full matrix least-squares methods with anisotropic thermal parameters for non-hydrogen atoms on F^2^. All hydrogen atoms were located in the calculated sites and included in the final refinement in the riding model approximation with displacement parameters derived from the parent atoms to which they were bonded (*U*_iso_(H) = 1.2*U*eq).

### Crystal data

**Table:**

C_10_H_8_N_2_·C_2_H_4_N_4_S	*F*(000) = 568
*M**_r_* = 272.34	*D*_x_ = 1.374 Mg m^−^^3^
Monoclinic, *P*2_1_/*c*	Mo *K*α radiation, λ = 0.71073 Å
*a* = 13.5151 (12) Å	Cell parameters from 2519 reflections
*b* = 6.9680 (5) Å	θ = 2.9–27.9°
*c* = 16.5529 (14) Å	µ = 0.24 mm^−^^1^
β = 122.385 (2)°	*T* = 298 K
*V* = 1316.39 (19) Å^3^	Block, yellow
*Z* = 4	0.40 × 0.30 × 0.21 mm

### Data collection

**Table:**

Bruker SMART CCD area-detector diffractometer	2315 independent reflections
Radiation source: fine-focus sealed tube	1725 reflections with *I* > 2σ(*I*)
Graphite monochromator	*R*_int_ = 0.034
phi and ω scans	θ_max_ = 25.0°, θ_min_ = 3.2°
Absorption correction: multi-scan (*SADABS*; Bruker, 2007)	*h* = −16→16
*T*_min_ = 0.910, *T*_max_ = 0.951	*k* = −8→6
6507 measured reflections	*l* = −19→15

### Refinement

**Table:**

Refinement on *F*^2^	Secondary atom site location: difference Fourier map
Least-squares matrix: full	Hydrogen site location: inferred from neighbouring sites
*R*[*F*^2^ > 2σ(*F*^2^)] = 0.040	H-atom parameters constrained
*wR*(*F*^2^) = 0.114	*w* = 1/[σ^2^(*F*_o_^2^) + (0.0451*P*)^2^ + 0.5763*P*] where *P* = (*F*_o_^2^ + 2*F*_c_^2^)/3
*S* = 1.09	(Δ/σ)_max_ = 0.001
2315 reflections	Δρ_max_ = 0.40 e Å^−^^3^
173 parameters	Δρ_min_ = −0.29 e Å^−^^3^
0 restraints	Extinction correction: *SHELXL97* (Sheldrick, 2008), Fc^*^=kFc[1+0.001xFc^2^λ^3^/sin(2θ)]^-1/4^
Primary atom site location: structure-invariant direct methods	Extinction coefficient: 0.028 (2)

### Special details

**Table:**

Geometry. All e.s.d.'s (except the e.s.d. in the dihedral angle between two l.s. planes) are estimated using the full covariance matrix. The cell e.s.d.'s are taken into account individually in the estimation of e.s.d.'s in distances, angles and torsion angles; correlations between e.s.d.'s in cell parameters are only used when they are defined by crystal symmetry. An approximate (isotropic) treatment of cell e.s.d.'s is used for estimating e.s.d.'s involving l.s. planes.
Refinement. Refinement of *F*^2^ against ALL reflections. The weighted *R*-factor *wR* and goodness of fit *S* are based on *F*^2^, conventional *R*-factors *R* are based on *F*, with *F* set to zero for negative *F*^2^. The threshold expression of *F*^2^ > σ(*F*^2^) is used only for calculating *R*-factors(gt) *etc*. and is not relevant to the choice of reflections for refinement. *R*-factors based on *F*^2^ are statistically about twice as large as those based on *F*, and *R*- factors based on ALL data will be even larger.

### Fractional atomic coordinates and isotropic or equivalent isotropic displacement parameters (Å^2^)

**Table:**

	*x*	*y*	*z*	*U*_iso_*/*U*_eq_	
N1	0.54234 (15)	0.3890 (3)	0.81338 (12)	0.0356 (5)	
H1	0.4953	0.3948	0.7523	0.043*	
N2	0.69266 (15)	0.3516 (3)	0.95219 (12)	0.0377 (5)	
H2	0.7632	0.3273	0.9982	0.045*	
N3	0.60324 (16)	0.3979 (3)	0.96667 (13)	0.0377 (5)	
N4	0.40154 (16)	0.4607 (3)	0.85462 (14)	0.0487 (6)	
H4A	0.3859	0.4756	0.8982	0.058*	
H4B	0.3469	0.4722	0.7953	0.058*	
N5	1.08698 (16)	0.6753 (3)	0.87403 (13)	0.0420 (5)	
N6	0.62173 (17)	0.6303 (3)	0.38615 (13)	0.0451 (5)	
S1	0.74194 (6)	0.31020 (11)	0.81472 (5)	0.0535 (3)	
C1	0.65892 (18)	0.3483 (3)	0.86103 (15)	0.0345 (5)	
C2	0.51320 (19)	0.4188 (3)	0.87965 (15)	0.0342 (5)	
C3	0.9754 (2)	0.6751 (4)	0.84854 (17)	0.0480 (6)	
H3	0.9595	0.6770	0.8967	0.058*	
C4	0.8821 (2)	0.6721 (4)	0.75504 (16)	0.0438 (6)	
H4	0.8058	0.6755	0.7414	0.053*	
C5	0.90223 (18)	0.6640 (3)	0.68118 (15)	0.0310 (5)	
C6	1.01851 (19)	0.6645 (4)	0.70791 (16)	0.0417 (6)	
H6	1.0375	0.6606	0.6616	0.050*	
C7	1.1056 (2)	0.6709 (4)	0.80326 (17)	0.0460 (6)	
H7	1.1828	0.6721	0.8191	0.055*	
C8	0.7294 (2)	0.6805 (4)	0.41216 (18)	0.0546 (7)	
H8	0.7437	0.7076	0.3643	0.065*	
C9	0.8213 (2)	0.6949 (4)	0.50561 (17)	0.0509 (7)	
H9	0.8948	0.7324	0.5193	0.061*	
C10	0.80481 (18)	0.6537 (3)	0.57918 (15)	0.0328 (5)	
C11	0.6921 (2)	0.6022 (4)	0.55197 (16)	0.0444 (6)	
H11	0.6752	0.5737	0.5982	0.053*	
C12	0.6055 (2)	0.5932 (4)	0.45693 (17)	0.0495 (6)	
H12	0.5306	0.5589	0.4410	0.059*	

### Atomic displacement parameters (Å^2^)

**Table:**

	*U*^11^	*U*^22^	*U*^33^	*U*^12^	*U*^13^	*U*^23^
N1	0.0317 (10)	0.0518 (12)	0.0198 (9)	−0.0015 (8)	0.0114 (8)	−0.0012 (8)
N2	0.0271 (10)	0.0541 (12)	0.0266 (10)	0.0016 (8)	0.0109 (8)	−0.0015 (8)
N3	0.0343 (10)	0.0523 (12)	0.0273 (10)	0.0052 (9)	0.0170 (9)	0.0023 (9)
N4	0.0319 (11)	0.0795 (16)	0.0330 (11)	0.0092 (10)	0.0162 (9)	0.0050 (10)
N5	0.0352 (11)	0.0506 (13)	0.0296 (10)	−0.0007 (9)	0.0102 (9)	0.0000 (9)
N6	0.0417 (12)	0.0526 (13)	0.0265 (10)	0.0024 (9)	0.0086 (9)	−0.0008 (9)
S1	0.0467 (4)	0.0750 (5)	0.0509 (4)	−0.0104 (3)	0.0342 (4)	−0.0130 (3)
C1	0.0324 (12)	0.0398 (13)	0.0284 (12)	−0.0063 (10)	0.0145 (10)	−0.0040 (9)
C2	0.0344 (12)	0.0412 (13)	0.0264 (12)	−0.0010 (10)	0.0157 (10)	0.0019 (10)
C3	0.0418 (14)	0.0716 (18)	0.0290 (13)	−0.0008 (12)	0.0178 (11)	−0.0008 (12)
C4	0.0313 (12)	0.0660 (17)	0.0316 (13)	−0.0017 (11)	0.0152 (11)	0.0021 (11)
C5	0.0308 (11)	0.0312 (12)	0.0268 (11)	−0.0009 (9)	0.0125 (9)	0.0011 (9)
C6	0.0348 (13)	0.0588 (16)	0.0328 (13)	−0.0009 (11)	0.0190 (11)	−0.0007 (11)
C7	0.0275 (12)	0.0640 (17)	0.0391 (14)	−0.0004 (11)	0.0129 (11)	0.0008 (12)
C8	0.0579 (17)	0.0742 (19)	0.0285 (13)	−0.0081 (14)	0.0211 (13)	0.0033 (12)
C9	0.0401 (14)	0.0761 (19)	0.0322 (13)	−0.0137 (13)	0.0165 (12)	0.0004 (12)
C10	0.0333 (12)	0.0336 (12)	0.0266 (11)	0.0004 (9)	0.0128 (10)	−0.0005 (9)
C11	0.0353 (13)	0.0659 (17)	0.0298 (12)	−0.0015 (11)	0.0160 (11)	−0.0011 (11)
C12	0.0324 (13)	0.0705 (18)	0.0352 (14)	−0.0017 (12)	0.0113 (11)	−0.0038 (12)

### Geometric parameters (Å, º)

**Table:**

N1—C1	1.362 (3)	C3—H3	0.9300
N1—C2	1.365 (3)	C4—C5	1.388 (3)
N1—H1	0.8600	C4—H4	0.9300
N2—C1	1.322 (3)	C5—C6	1.386 (3)
N2—N3	1.390 (2)	C5—C10	1.486 (3)
N2—H2	0.8600	C6—C7	1.374 (3)
N3—C2	1.304 (3)	C6—H6	0.9300
N4—C2	1.365 (3)	C7—H7	0.9300
N4—H4A	0.8600	C8—C9	1.374 (3)
N4—H4B	0.8600	C8—H8	0.9300
N5—C7	1.323 (3)	C9—C10	1.380 (3)
N5—C3	1.331 (3)	C9—H9	0.9300
N6—C8	1.324 (3)	C10—C11	1.384 (3)
N6—C12	1.328 (3)	C11—C12	1.370 (3)
S1—C1	1.685 (2)	C11—H11	0.9300
C3—C4	1.376 (3)	C12—H12	0.9300
			
C1—N1—C2	107.97 (17)	C6—C5—C4	116.2 (2)
C1—N1—H1	126.0	C6—C5—C10	121.74 (19)
C2—N1—H1	126.0	C4—C5—C10	122.03 (19)
C1—N2—N3	113.61 (18)	C7—C6—C5	119.6 (2)
C1—N2—H2	123.2	C7—C6—H6	120.2
N3—N2—H2	123.2	C5—C6—H6	120.2
C2—N3—N2	102.64 (17)	N5—C7—C6	124.5 (2)
C2—N4—H4A	120.0	N5—C7—H7	117.8
C2—N4—H4B	120.0	C6—C7—H7	117.8
H4A—N4—H4B	120.0	N6—C8—C9	123.9 (2)
C7—N5—C3	116.07 (19)	N6—C8—H8	118.0
C8—N6—C12	115.9 (2)	C9—C8—H8	118.0
N2—C1—N1	104.02 (18)	C8—C9—C10	120.2 (2)
N2—C1—S1	127.97 (17)	C8—C9—H9	119.9
N1—C1—S1	127.98 (16)	C10—C9—H9	119.9
N3—C2—N1	111.74 (18)	C9—C10—C11	115.8 (2)
N3—C2—N4	125.82 (19)	C9—C10—C5	121.9 (2)
N1—C2—N4	122.42 (19)	C11—C10—C5	122.21 (19)
N5—C3—C4	123.8 (2)	C12—C11—C10	120.0 (2)
N5—C3—H3	118.1	C12—C11—H11	120.0
C4—C3—H3	118.1	C10—C11—H11	120.0
C3—C4—C5	119.8 (2)	N6—C12—C11	124.1 (2)
C3—C4—H4	120.1	N6—C12—H12	117.9
C5—C4—H4	120.1	C11—C12—H12	117.9
			
C1—N2—N3—C2	1.2 (3)	C3—N5—C7—C6	−0.6 (4)
N3—N2—C1—N1	−1.6 (2)	C5—C6—C7—N5	0.5 (4)
N3—N2—C1—S1	176.54 (17)	C12—N6—C8—C9	0.1 (4)
C2—N1—C1—N2	1.4 (2)	N6—C8—C9—C10	0.8 (4)
C2—N1—C1—S1	−176.79 (18)	C8—C9—C10—C11	−1.0 (4)
N2—N3—C2—N1	−0.3 (2)	C8—C9—C10—C5	178.9 (2)
N2—N3—C2—N4	178.2 (2)	C6—C5—C10—C9	−17.5 (3)
C1—N1—C2—N3	−0.7 (3)	C4—C5—C10—C9	163.0 (2)
C1—N1—C2—N4	−179.3 (2)	C6—C5—C10—C11	162.4 (2)
C7—N5—C3—C4	−0.6 (4)	C4—C5—C10—C11	−17.1 (3)
N5—C3—C4—C5	1.7 (4)	C9—C10—C11—C12	0.5 (4)
C3—C4—C5—C6	−1.6 (3)	C5—C10—C11—C12	−179.4 (2)
C3—C4—C5—C10	177.8 (2)	C8—N6—C12—C11	−0.7 (4)
C4—C5—C6—C7	0.6 (3)	C10—C11—C12—N6	0.4 (4)
C10—C5—C6—C7	−178.9 (2)		

### Hydrogen-bond geometry (Å, º)

**Table:**

*D*—H···*A*	*D*—H	H···*A*	*D*···*A*	*D*—H···*A*
N1—H1···N6^i^	0.86	1.98	2.828 (3)	171
N2—H2···N5^ii^	0.86	2.00	2.829 (3)	163
N4—H4*A*···N3^iii^	0.86	2.33	3.151 (3)	159
N4—H4*B*···S1^iv^	0.86	2.82	3.424 (2)	129

Symmetry codes: (i) −*x*+1, −*y*+1, −*z*+1; (ii) −*x*+2, −*y*+1, −*z*+2; (iii) −*x*+1, −*y*+1, −*z*+2; (iv) −*x*+1, *y*+1/2, −*z*+3/2.
